# Innate immune response to a H3N2 subtype swine influenza virus in newborn porcine trachea cells, alveolar macrophages, and precision-cut lung slices

**DOI:** 10.1186/1297-9716-45-42

**Published:** 2014-04-09

**Authors:** Mario Delgado-Ortega, Sandrine Melo, Darsaniya Punyadarsaniya, Christelle Ramé, Michel Olivier, Denis Soubieux, Daniel Marc, Gaëlle Simon, Georg Herrler, Mustapha Berri, Joëlle Dupont, François Meurens

**Affiliations:** 1 INRA, Infectiologie et Santé Publique, 37380 Nouzilly, France; 2 Université François Rabelais, UMR1282 Infectiologie et Santé Publique, 37000 Tours, France; 3 Veterinary Medicine Faculty, Mahanakorn University of technology, Bangkok, Thailand; 4 INRA, Physiologie de la Reproduction et des Comportements, 37380 Nouzilly, France; 5 Anses, Ploufragan/Plouzané Laboratory, Swine Virology Immunology Unit, BP 53, 22440 Ploufragan, France; 6 European University of Brittany, 35000 Rennes, France; 7 Institut für Virologie, Tierärztliche Hochschule Hannover, Hanover, Germany; 8 Vaccine and Infectious Disease Organization-InterVac, University of Saskatchewan, 120 Veterinary Road, S7N 5E3 Saskatoon, Saskatchewan, Canada

## Abstract

Viral respiratory diseases remain of major importance in swine breeding units. Swine influenza virus (SIV) is one of the main known contributors to infectious respiratory diseases. The innate immune response to swine influenza viruses has been assessed in many previous studies. However most of these studies were carried out in a single-cell population or directly in the live animal, in all its complexity. In the current study we report the use of a trachea epithelial cell line (newborn pig trachea cells – NPTr) in comparison with alveolar macrophages and lung slices for the characterization of innate immune response to an infection by a European SIV of the H3N2 subtype. The expression pattern of transcripts involved in the recognition of the virus, interferon type I and III responses, and the host-response regulation were assessed by quantitative PCR in response to infection. Some significant differences were observed between the three systems, notably in the expression of type III interferon mRNA. Then, results show a clear induction of JAK/STAT and MAPK signaling pathways in infected NPTr cells. Conversely, PI3K/Akt signaling pathways was not activated. The inhibition of the JAK/STAT pathway clearly reduced interferon type I and III responses and the induction of SOCS1 at the transcript level in infected NPTr cells. Similarly, the inhibition of MAPK pathway reduced viral replication and interferon response. All together, these results contribute to an increased understanding of the innate immune response to H3N2 SIV and may help identify strategies to effectively control SIV infection.

## Introduction

Viral respiratory diseases are still a major health issue in pigs reared under confined conditions on intensive breeding farms worldwide. Currently the most common viral pathogens are porcine reproductive and respiratory syndrome virus (PRRSV), swine influenza virus (SIV), pseudorabies virus, and porcine circovirus type 2 [1-3]. In the field, these viruses are usually found in association with each other or with bacteria such as *Actinobacillus spp.*, *Bordetella bronchiseptica*, *Haemophilus parasuis*, *Mycoplasma hyopneumoniae*, *Pasteurella multocida*, and *Streptococcus suis*[1,3-5]. In particular, influenza A viruses are a major cause of acute respiratory disease on pig breeding farms [6]. Usually, the disease is characterized by depression, loss of appetite, abdominal breathing, tachypnea, fever, and less often, coughing. Morbidity associated with the virus is high and mortality is often very low [6]. Influenza A viruses are classified into subtypes based on the antigenicity of their hemagglutinin (HA) and their neuraminidase (NA) surface proteins [6]. In pigs, three subtypes are described worldwide: H1N1, H1N2, and H3N2 [7,8], however genetic lineage may vary within each subtype depending on the geographical location (North America, Europe, and Asia). Viruses of the three subtypes have been reported frequently in European pigs, often associated with clinical disease [9,10].

The innate immune response is crucial in the fight against respiratory viruses. It controls viral invasion and replication while the adaptive response is effective for viral clearance through the activity of adaptive immune cells such as lymphocytes [11,12]. Among innate immune cells, epithelial cells, alveolar macrophages, and dendritic cells provide the first line of defense [11-15]. They rapidly recognize pathogens through pattern recognition receptors (PRRs) such as Toll-like receptors (TLRs), intracellular viral sensors such as retinoic acid-inducible gene I (RIG-I) and melanoma differentiation-associated gene 5 (MDA5), and the nucleotide-binding domain, leucine-rich repeat-containing proteins (NLRs) [16,17]. The innate immune response to SIV has been assessed in many previous studies (for review see [6,16]). However, most of these studies were carried out in a single-cell population, sometimes isolated from other species, or directly in the pig in all its complexity making the establishment of clear conclusions difficult.

Newborn porcine trachea (NPTr) cells [18], porcine alveolar macrophages (PAMs), and precision-cut lung slices (PCLS) may constitute informative and complementary models to study the innate immune response to SIV. Respiratory epithelial cells and PAMs are the main target of the virus in vivo and have been successfully used for in vitro infection studies [6,15,16,19]. PCLS have previously been used for infection studies in birds [20], cattle [21], and pigs [22,23]. The PCLS culture system has several advantages over other systems: the slices can be obtained in large numbers and the general architecture of the tissue is preserved. Thus, differentiated epithelial cells, which are the main target cells of SIV, are maintained in situ and the slices remain viable for more than 7 days [23]. Thus, NPTr cells, PAMs and PCLS together enable the study of host/pathogen interactions in single-cell type populations as well as a multi-cellular tissue, granting more accurate analysis of the contribution of epithelial cells and macrophages to the global disease response. Use of lung explants (ie PCLS) to study SIV infection has only been reported in a few cases [23-27]. However, the focus of these studies was not the characterization of the innate immune response to the virus. Here, we report the use of NPTr cells in comparison to PAMs and PCLS for the study of the innate immune response to a European H3N2 SIV. Transcripts involved in the recognition of viral patterns, interferon (IFN) type I and III responses, and regulation of the host response (suppressor of cytokine signaling, SOCS) were assessed by qPCR for their expression in response to the infection at different time points. Some significant differences were observed between the three systems, notably in the expression of type III IFN mRNA. For instance, in NPTr cells and PCLS we observed mostly IFNβ and IFNλ1 transcript expression in response to the virus while in PAMs, IFN type III was not significantly induced. The involvement of different signaling pathways such as janus kinase (JAK)/signal transducer and activator of transcription (STAT), mitogen-activated protein kinase (MAPK)/extracellular signal-regulated kinase (ERK1/2), and phosphatidylinositide 3-kinase (PI3K)/protein kinase B (Akt) was also evaluated in NPTr cells. Our results show a clear induction of JAK/STAT and MAPK (ERK1/2, p38) signaling pathways while the H3N2 SIV did not induce PI3K/Akt activation in infected NPTr cells. The inhibition of the JAK/STAT pathway using JAK Inhibitor I (420099) in infected NPTR cells had a clear impact on the response of IFN types I and III and the induction of SOCS1 at the transcript level. All together, our data contribute to an increased understanding of the innate immune response of pigs to SIV.

## Materials and methods

### Ethics statement

Pigs used for this research were kept in the clinic for swine and small ruminants for demonstration and veterinary student training (approval number 33.9-42502-05-09A627) or were obtained from the INRA experimental unit (Nouzilly, France). A total of 12 eight-week-old pigs (German Landrace and Large White) were used. Pigs were healthy and showed no clinical symptoms or serological evidence of influenza and other respiratory or systemic diseases. All studies were carried out in accordance with the recommendations of the European Convention for the Protection of Vertebrate Animals used for Experimental and Other Scientific Purposes (European Treaty Series, nos. 123 [28] and 170 [29] and in accordance with the guidelines of the Institutional Animal Care and Use committee at INRA (France). The protocol was approved by the national and local permitting authorities (animal welfare officer of the University of Veterinary Medicine, Lower Saxony State Office for Consumer Protection and Food Safety). All experimental measurements were in accordance with the requirements of the national animal welfare law. Euthanasia and tissue sampling were performed under sodium pentobarbital anesthesia, and all efforts were made to minimize suffering of the animals.

### Epithelial cell line culture

The newborn pig trachea (NPTr, purchased from *Istituto Zooprofilattico Sperimentale, della Lombardia e dell’Emilia Romagna*, Brescia, Italy) cells [18] (between 30 and 50 passages) were cultured in Dulbecco’s modified Eagle medium (DMEM) (Invitrogen, Cergy Pontoise, France) supplemented with 10% fetal calf serum (FCS) (Sigma-Aldrich, Saint-Quentin, France), 20 IU/mL of penicillin, and 20 mg/mL of streptomycin (Invitrogen). Cells were plated onto sterile 24-well plastic plates (Greiner bio-one, Courtabœuf, France) and incubated at 37 °C in a 5% CO_2_ humidified atmosphere. Sub-passages were made when cells reached 100% confluence.

### Porcine alveolar macrophages

Porcine alveolar macrophages (PAMs) were obtained from broncho-alveolar lavage (BAL) fluid from a total of 7 eight-week-old pigs and maintained in DMEM supplemented with 2% FCS, 14 mM 4-(2-hydroxyethyl)-1-piperazineethanesulfonic acid (HEPES) (Gibco-Life Technologies SAS, Saint-Aubin, France), 0.1 mM non-essential amino acids (Gibco), 100 μg/mL gentamycin (Gibco), 20 IU/mL of penicillin, and 20 mg/mL of streptomycin (Invitrogen). Red blood cells were eliminated and PAMs were isolated by multiple washes and low-speed centrifugations. PAMs were plated onto sterile 24-well plastic plates (Greiner bio-one) and incubated at 37 °C and 5% CO_2_ for 3 h to allow macrophages to adhere. Non-adherent cells were eliminated. PAMs represented > 90% of cells in broncho-alveolar lavage fluid as previously reported [30].

### Precision-cut lung slices

Precision-cut lung slices (PCLS) were prepared from the lungs of 5 eight-week-old pigs (minimum one slice/pig for each time point). Immediately after euthanasia, lungs were carefully removed and the left cranial, middle, and caudal lobes were filled with 37 °C low-gelling temperature agarose (GERBU Biotechnik GmbH, Gaiberg, Germany) followed by polymerization on ice. Tissue was excised in cylindrical portions (8-mm tissue-coring tool) and roughly 200 slices/pig approximately 250 μm thick were prepared by using a Krumdieck tissue slicer (model MD4000-01, TSE systems, Chesterfield, MO, USA) with a cycle speed of 60 slices/min. PCLS were incubated in 1 mL of Roswell Park Memorial Institute medium (RPMI) 1640 medium (Gibco), supplemented with 1% antibiotic/antimycotic liquid (100X Antibiotic-Antimycotic, Gibco), 1 μg/mL clotrimazole (Sigma–Aldrich), 10 μg/mL enrofloxacin (Bayer Animal Health, Germany), and 80 μg/mL kanamycin (Gibco) in a sterile 24-well plate at 37 °C and 5% CO_2_. The medium was changed every hour during the first 4 h and once after 24 h, prior to infection. Viability was analyzed by observing ciliary activity under a light microscope (Olympus CKX31, Tokyo, Japan). In selected samples, slices were analyzed for bronchoconstriction by addition of 10^-4^ M methacholine (acetyl-ß-methylcholine chloride, Sigma-Aldrich), as previously described [31].

### Virus strain and propagation

The swine influenza virus strain A/Swine/Bissendorf/IDT1864/2003 of the H3N2 subtype was isolated from a pig with acute respiratory syndrome in a German herd and kindly provided by Ralf Dürrwald, IDT Biologika GmbH (Dessau-Rosslau, Germany). It was further propagated in the porcine epithelial NPTr cell line by infection at a low multiplicity of infection (MOI) (0.001 plaque forming unit/cell). NPTr cell line has been demonstrated suitable for the culture of SIV [18]. Forty-eight hours post-infection, supernatants were clarified by low-speed centrifugation (2000 × *g*, 5 min), then stored at -80 °C until use. The viral titer of this stock reached 3.3 × 10^8^ plaque forming units (pfu)/mL as determined by a plaque assay on NPTr cells, as described previously [32].

### Virus infection

NPTr cells and PAMs were seeded onto sterile 24-well plates at 2-4 × 10^5^ cells per well and infected with H3N2 at an MOI of 1 (enabling, according to the Poisson distribution, the infection of 63.2% of the cells with at least one single particle). After 1 h of incubation at 37 °C and 5% CO_2_ to allow virus adsorption, cells were washed once with phosphate buffered saline (PBS) and further maintained at 37 °C and 5% CO_2_ in 1 mL of medium. For PCLS infection, the procedure was nearly identical except that 10^6^ pfu of virus/slice were used since it was not possible to determine the number of target cells in a single slice. The cells and lung slices were incubated at 37 °C and 5% CO_2_ for the different time points before collection for RNA extraction or staining.

### Real-time PCR assays and validation of reference genes

NPTr cells and PAMs were lysed in RLT lysis buffer (Qiagen, Courtabœuf, France). Precision-cut lung slices were lysed and homogenized in Trizol reagent (Invitrogen) using ceramic beads (BioSpec Products, OK, USA) and the FastPrep FP120 cell disrupter (Qbiogene, Illkirch, France). Total RNA was isolated using RNeasy Mini Kit (Qiagen) following the manufacturer’s recommendations. Quantitative real-time PCR (qPCR) was performed using cDNA synthesized as previously described [33,34]. Primers to assess transcript expression and viral replication were already published or were designed using Clone Manager 9 (Scientific & Educational Software, Cary, NC, USA) and were purchased from Eurogentec (Liège, Belgium) (Table 1). Diluted cDNA (4×) was combined with primer/probe sets and IQ SYBR Green Supermix (Bio-Rad, Hercules, CA, USA) according to the manufacturer’s recommendations. The qPCR conditions were 98 °C for 30 s, followed by 37 cycles with denaturation at 95 °C for 15 s and annealing/elongation for 30 s (annealing temperature, Table 1). Real time assays were run on a Bio-Rad Chromo 4 (Bio-Rad, Hercules, CA, USA). The specificity of the qPCR reactions was assessed by analyzing the melting curves of the products and verifying the amplicon sizes. To minimize sample variation we used identical amounts of high quality RNA from cells and tissue. The RNA quality was assessed by capillary electrophoresis (Agilent 2100 Bioanalyzer, Agilent Technologies, Massy, France) and RNA integrity numbers (RIN) were calculated. RIN were always ≥ 8.1 for tissue and ≥ 9.5 for cells. Samples were normalized internally by simultaneously using the average Cycle quantification (*Cq*) of the three most suitable reference genes in each sample to avoid any artefact of variation in the target gene. These three most suitable reference genes were selected among eight commonly used reference genes. The genes included beta-actin (ActB), beta-2-microglobulin (B2MI), glyceraldehyde-3-phosphate dehydrogenase (GAPDH), hydroxymethylbilane synthase (HMBS), hypoxanthine phosphoribosyltransferase-1 (HPRT-1), ribosomal protein L-19 (RPL-19), succinate dehydrogenase complex subunit A (SDHA) and TATA box binding protein-1 (TPB-1) (Table 1). The stability of these reference genes was investigated simultaneously in control and infected (1 to 24 h post-infection) NPTR cells, PAMs, and PCLS using the geNorm application [31,35,36]. The threshold for eliminating a gene was M ≥ 1 for tissue samples and M ≥ 0.5 for cells as recommended [36]. The correlation coefficients of the standard curves were > 0.995 and the concentration of the test samples was calculated from the standard curves, according to the formula y = -M*Cq + B, where M is the slope of the curve, *Cq* the first positive second derivative maximum of amplification curve calculated using PCR Miner [37] and B the y-axis intercept. All qPCRs displayed efficiency between 90% and 110%. Expression data were expressed as relative values after Genex macro analysis (Bio-Rad, Hercules, CA, USA) [35].

**Table 1:** Primer sequences, annealing temperatures of primer sets (°C), expected PCR fragment sizes (bp) and accession numbers or references

**Primer name**	**Primer sequence**	**Annealing temperature (s) (°C)**	**PCR product (bp)**	**Accession number or reference**
***ActB***	CACGCCATCCTGCGTCTGGA	63	100	[38]
*Beta actin*	AGCACCGTGTTGGCGTAGAG			
***B2MI***	CAAGATAGTTAAGTGGGATCGAGAC	58	161	[38]
*Beta-2-microgobulin*	TGGTAACATCAATACGATTTCTGA			
***CISH***	GGGAATCTGGCTGGTATTGG	62	126	BE014034
*Cytokine-inducible SH2-containing protein*	CCGACAGTGTGAACAGGTAG			
***GAPDH***	CTTCACGACCATGGAGAAGG	63	170	AF017079
*Glyceraldehyde-3-phosphate dehydrogenase*	CCAAGCAGTTGGTGGTACAG			
***HMBS-2***	AGGATGGGCAACTCTACCTG	58	83	[38]
*Hydroxymethylbilane synthase 2*	GATGGTGGCCTGCATAGTCT			
***HPRT-1***	GGACTTGAATCATGTTTGTG	60	91	[38]
*Hypoxanthine phosphoribosyltransferase 1*	CAGATGTTTCCAAACTCAAC			
***IL-1β***	AGAAGAGCCCATCGTCCTTG	62	139	NM_001005149
*Interleukine 1 beta*	GAGAGCCTTCAGCTCATGTG			
***IL-6***	ATCAGGAGACCTGCTTGATG	62	177	NM_214399
*Interleukine 6*	TGGTGGCTTTGTCTGGATTC			
***IL-8***	TCCTGCTTTCTGCAGCTCTC	62	100	NM_213867
*Interleukine 8*	GGGTGGAAAGGTGTGGAATG			
***IFNα (1-17)***	GGCTCTGGTGCATGAGATGC	64	197	[39]
*Interferon alpha (Type I)*	CAGCCAGGATGGAGTCCTCC			
***IFNβ***	ATGTCAGAAGCTCCTGGGACAGTT	62	246	[39]
*Interferon beta (Type I)*	AGGTCATCCATCTGCCCATCAAGT			
***IFNγ***	GCTCTGGGAAACTGAATGAC	60	167	NM_213948
*Interferon gamma (Type II)*	TCTCTGGCCTTGGAACATAG			
***IFNλ1/IL-29A***	GAGGCTGAGCTAGACTTGAC	60	115	NM_001142837
*Interferon lambda1 (Type III)*	CCTGAAGTTCGACGTGGATG			
***IFNλ3/IL-28B***	GGCTCCTTGGCGAACTCATC	62	173	GQ996936
*Interferon lambda3 (Type III)*	TCCTTCTTCTGGGCCTCCTG			
***iNOS***	GAGAGGCAGAGGCTTGAGAC	62	178	BI344008
*Inducible nitric oxide synthase*	TGGAGGAGCTGATGGAGTAG			
***Mx1***	AGTGTCGGCTGTTTACCAAG	60	151	NM_214061
*Myxovirus resistance 1*	TTCACAAACCCTGGCAACTC			
***Mx2***	CCGACTTCAGTTCAGGATGG	62	156	AB258432
*Myxovirus resistance 2*	ACAGGAGACGGTCCGTTTAC			
***OAS1***	CCCTGTTCGCGTCTCCAAAG	64	303	NM_214303
*2′-5′-Oligoadenylate synthetase 1*	GCGGGCAGGACATCAAACTC			
***SIV M protein***	AGATGAGTCTTCTAACCGAGGTCG	62	100	[40]
	TGCAAAAACATCTTCAAGTCTCTG			
***PKR***	GACATCCAAAGCAGCTCTCC	62	365	NM_214319
*Protein Kinase RNA-dependent*	CGCTCTACCTTCTCGCAATC			
***RPL-19***	AACTCCCGTCAGCAGATCC	60	147	[41]
*Ribosomal protein L19*	AGTACCCTTCCGCTTACCG			
***RIG-I***	CGACATTGCTCAGTGCAATC	60	126	NM_213804
*Retinoic acid-inducible gene I*	TCAGCGTTAGCAGTCAGAAG			
***SDHA***	CTACAAGGGGCAGGTTCTGA	58	141	[38]
*Succinate dehydrogenase complex subunit A*	AAGACAACGAGGTCCAGGAG			
***SOCS1***	CGCCCTCAGTGTGAAGATGG	62	110	EW101597
*Suppressor of cytokine signaling 1*	GCTCGAAGAGGCAGTCGAAG			
***SOCS3***	CAGCTCCAAGAGCGAGTACC	61	179	NM_001123196
*Suppressor of cytokine signaling 3*	TGACGCTGAGCGTGAAGAGG			
***TBP-1***	AACAGTTCAGTAGTTATGAGCCAGA	60	153	[38]
*TATA box binding protein 1*	AGATGTTCTCAAACGCTTCG			
***TLR3***	CCTGCATTCCAGAAGTTGAG	60	152	NM_001097444
*Toll Like Receptor 3*	TGAGGTGGAGTATTGCAGAG			
***TLR7***	TCAGCTACAACCAGCTGAAG	60	140	NM_001097434
*Toll Like Receptor 7*	CAGATGTCGCAACTGGAAAG			
***TLR8***	AGCGCGGGAGGAGTATTGTG	62	118	NM_214187
*Toll Like Receptor 8*	GCCAGGGCAGCCAACATAAC			
***TNFα***	CCAATGGCAGAGTGGGTATG	62	116	X54859
*Tumor Necrosis Factor alpha*	TGAAGAGGACCTGGGAGTAG			

Reference genes are in italic.

### Cryosections and immunofluorescence analysis

Infected and non-infected PCLS were mounted on small pieces of filter paper with tissue-freezing medium (Jung, Heidelberg, Germany), then frozen in liquid nitrogen and kept at -80 °C prior to cutting. Ten μm-thick slices were cut by a cryotome (Reichert-Jung, Nußloch, Germany). The sections were dried overnight at room temperature and then kept frozen at -20 °C until staining.

The sections were fixed with 3% paraformaldehyde for 20 min and permeabilized with 0.2% Triton X-100 for 5 min followed by three washing steps with PBS. All antibodies were diluted in 1% bovine serum albumin (Sigma-Aldrich) and incubated with the sections for 1 h at room temperature (RT) in a humid incubation chamber. After the final incubation step, the sections were washed three times with PBS and once with distilled water. The slices were embedded in Mowiol 4-88 resin (Sigma-Aldrich), covered by no. 1½ circular micro-cover glass (12 mm) (Electron Microscopy Sciences, Hatfield, PA, USA), and stored at 4 °C until examination under the confocal microscope. For detection of infected cells, a monoclonal antibody (IgG2a) against the influenza A virus nucleoprotein (NP) (Clone AA5H, AbDSeroTec MCA400, Düsseldorf, Germany) was used at a 1:750 dilution followed by incubation with an anti-mouse IgG (Sigma-Aldrich) secondary antibody. To visualize cilia, samples were treated with a Cy3-labeled monoclonal antibody recognizing beta-tubulin (dilution 1/600) (Sigma-Aldrich). Nuclei were stained by incubating sections for 15 min at 37 °C in 4′,6′-diamidino-2-phenylindole (DAPI) (Life Technologies Inc., Darmstadt, Germany).

### Western blotting

NPTr cells (2-4 × 10^5^ cells/well) were virus-infected at an MOI of 1, then incubated for 5, 10, 30, 60 or 240 min. Cells were then disrupted using the lysis buffer (10 mM Tris pH 7.4, 150 mM NaCl, 1 mM ethylene glycol tetraacetic acid, 1 mM ethylene diamine tetraacetic acid-EDTA, 1% (v/v) Triton ×-100, 0.5% NP-40), protease inhibitors (2 mM phenyl methyl sulfonyl fluoride-PMSF, 10 μg/mL leupeptin, 10 μg/mL aprotinin) and phosphatase inhibitors (100 mM sodium fluoride, 10 mM sodium pyrophosphate, 2 mM sodium orthovanadate) (Sigma-Aldrich) (Bio-Rad, Marnes-la-Coquette, France). Lysates were incubated on ice for 30 min and then centrifuged at 12 000 × *g* for 20 min at 4 °C. Equal amounts of proteins were separated using sodium dodecyl sulfate polyacrylamide gel electrophoresis (SDS-PAGE) and transferred onto a nitrocellulose membrane. Membranes were then incubated for 1 h at RT with Tris-buffered saline (TBS, 2 mM Tris-HCL, pH 8, 15 mM NaCl, pH 7.6), containing 5% non-fat dry milk powder (NFDMP) and 0.1% Tween-20 (Bio-Rad) to saturate non-specific sites. Then membranes were incubated overnight at 4 °C with appropriate primary antibodies (final dilution 1:1000, see Table 2) in TBS containing 0.1% Tween-20 and 5% NFDMP. The membranes were washed in TBS-0.1% Tween-20 and incubated for 2 h at RT with a horseradish peroxidase-conjugated secondary antibody (final dilution 1:10 000). After washing, proteins were detected by enhanced chemiluminescence (Western Lightning Plus-ECL, Perkin Elmer, Courtabœuf, France) using a G:Box SynGene (Ozyme, Saint-Quentin-en-Yvelines, France) with the GeneSnap software (Syngene UK, Cambridge, UK, release 7.09.17). Detected signals were quantified with the GeneTools software (Syngene UK, release 4.01.02). The results are expressed as the signal intensity in arbitrary units after normalization as indicated in the figure legends.

**Table 2:** Antibodies used for Western blotting

**Target**	**Antibody**	**Dilution**
**Phospho-Akt**	Rabbit polyclonal anti-phospho-Akt (Ser473)	1/1000
	#9271 (Ozyme)	
**Phospho-ERK1/2**	Rabbit monoclonal anti-phospho-p44/42 MAPK (Erk1/2)	1/1000
	(Thr202/Tyr204) (D13.14.4E) #4370 (Ozyme)	
**Phospho-JAK2**	Rabbit polyclonal anti-phospho-JAK2 (Tyr1007/1008)	1/1000
	#3771 (Ozyme)	
**Phospho-p38**	Rabbit monoclonal anti-phospho-p38 MAPK (Thr180/Tyr182)	1/1000
	(12 F8) #4631 (Ozyme)	
**Akt**	Rabbit monoclonal anti-Akt (11E7) #4685 (Ozyme)	1/1000
**ERK2**	Rabbit polyclonal anti-ERK2 (GTX27948) (tebu-bio)	1/1000
**JAK2**	Rabbit monoclonal anti-JAK2 (D2E12) #3230 (Ozyme)	1/1000
**p38**	Rabbit polyclonal anti-p38 MAPK (GTX50566) (tebu-bio)	1/1000

See also Additional files 1 and 2.

### Antibodies and chemical inhibitors

Antibodies to phospho-Akt (Ser 473), phospho-ERK1/2 (Thr 202/Tyr 204), phospho-JAK2 (Tyr 1007/1008), and phospho-p38 (Thr180/Tyr182) were purchased from Ozyme. Monoclonal and polyclonal antibodies to Akt, ERK2, p38, and JAK2 were obtained from Tebu Bio (Le Perray-en-Yvelines, France) and Ozyme. All antibodies were used at a 1:1000 dilution in assays. Stock solutions of pharmacological inhibitors such as inhibitor U0126 (inhibiting MEK1/2 and ERK1/2), p38/SAPK2-specific inhibitor SB 202190, and JAK Inhibitor I (420099) (Millipore, Molsheim, France) were all prepared as 1000-× concentrated stocks in dimethyl sulfoxide (DMSO), in order to ensure that the final concentration of DMSO in the culture medium did not exceed 0.1%. Starting one hour before infection, NPTr cells were treated for the whole procedure with each inhibitor at a concentration of 10 μM to block the signaling. The blockage of the cascades was verified at 30 min post-infection.

### Statistical analysis

Expression of mRNA in cells and tissues was expressed as relative values. All statistical analyses were done using Prism 5 computer software (Prism 5 for Windows; GraphPad Software, San Diego, CA, USA). One-Way ANOVA was used to detect differences between groups. To account for the non-normal distribution, all data were sorted by rank prior to performing the ANOVA. Tukey’s test was used to compare the means of the ranks among the groups. *P* values less than 0.05 were considered significant.

## Results

### Reference gene selection

**Figure 1:**
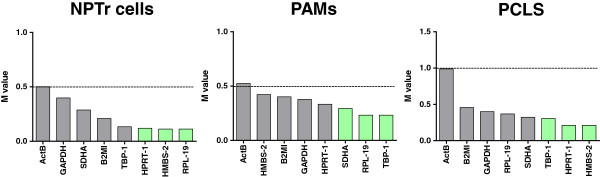
**Selected candidate reference genes and their expression stability.** The stability of the reference genes has been assessed in a mix of non-infected and infected newborn pig trachea cells (NPTr), porcine alveolar macrophages (PAMs) and precision-cut lung slices (PCLS) at the different time points. Gene expression stability of candidate reference genes was analyzed by the geNorm application. Threshold for eliminating a gene was ≥ 1.0 for PCLS and ≥ 0.5 for NPTr cells and PAMs. The three most stable reference genes are depicted in green.

In order to characterize the immune response against our strain of SIV in vivo and ex vivo using qPCR assays, validation of the three most stable reference genes in NPTr cells, PAMs, and PCLS was required (Figure 1). Eight previously described reference genes [33,38,42] were selected for assessment of their stability in our conditions. The selection was based on the M value using geNorm. The threshold was set at 0.5 for homogenous samples (e.g. NPTr cells and PAMs) and 1 for heterogeneous samples (PCLS) [36]. The geNorm analysis showed that HPRT-1, HMBS-2, and RPL-19 were the three most stable genes in NPTr cells with an M value of 0.119, 0.119, and 0.117, respectively (Figure 1). All of the reference genes tested had M values below the threshold, except ActB (0.501). In PAMs the situation was roughly similar and again ActB was the only gene with an M value above the elimination threshold (0.523); the three most stable genes were SDHA, RPL-19, and TBP-1 (0.293, 0.232, and 0.232, respectively) (Figure 1). The selected genes for PCLS were TBP-1, HPRT-1 and HMBS-2, having an M value of 0.305, 0.211, and 0.211, respectively (Figure 1). In the three systems, RPL19 was considered the most stable reference gene. ActB was the most variable reference gene with an M value equal or higher than the threshold.

### Viral replication in newborn pig trachea cells and alveolar macrophages

**Figure 2:**
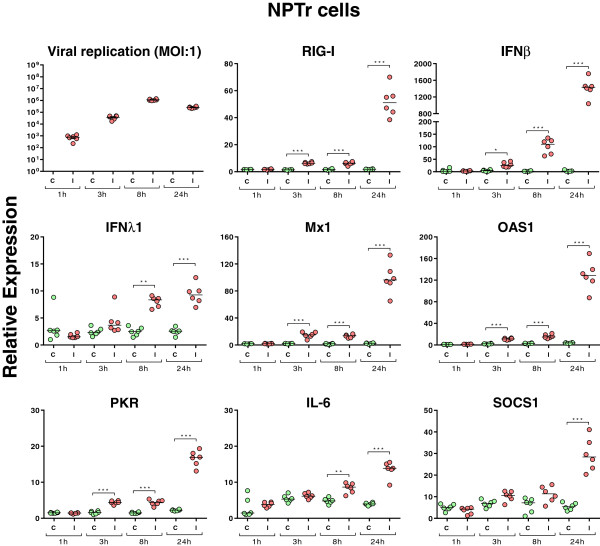
**Relative expression of M-viral RNA and of host transcripts in infected newborn pig trachea cells.** The NPTr cells were infected with the H3N2 SIV strain at different time points (1 h, 3 h, 8 h, and 24 h). Green dots stand for non-infected NPTr cells and red dots for infected cells (individual values (dots) and median value (bar), *n* = 6 wells per condition). Comparisons were made using one way ANOVA test and Tukey’s post-test. Differences were considered significant when *P* < 0.05 (*), *P* < 0.01 (**) or *P* < 0.001 (***).

**Figure 3:**
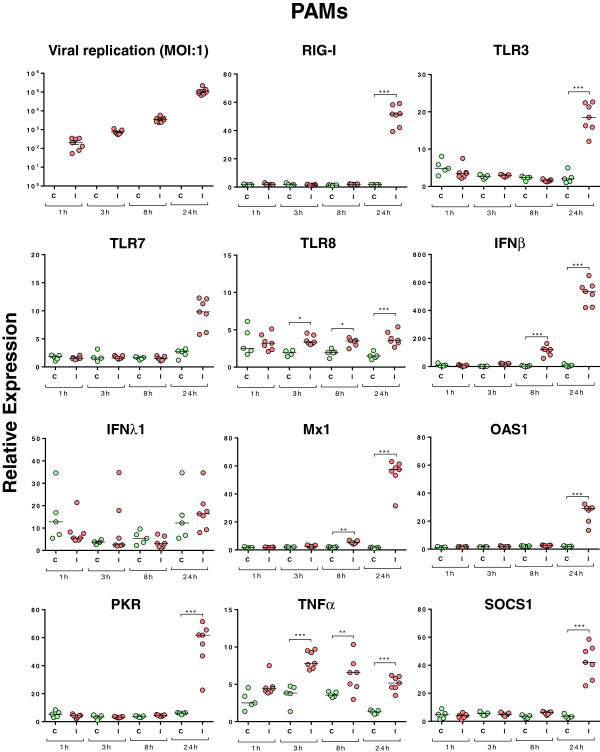
**Relative expression of various viral and host transcripts in infected porcine alveolar macrophages (PAMs).** The cells were infected with the H3N2 SIV strain at different time points (1 h, 3 h, 8 h, and 24 h). Green dots stand for non-infected PAMs and red dots for infected PAMs (each individual value (*n* = 7) represents the data obtained with PAMs prepared from one pig, bar represents the median value). Comparisons were made using one way ANOVA test and Tukey’s post-test. Differences were considered significant when *P* < 0.05 (*), *P* < 0.01 (**) or *P* < 0.001 (***).

In order to verify the capacity of our SIV strain to infect NPTr cells and PAMs, we assessed viral replication through quantification of the viral M-segment RNA (M-vRNA) in both cell types (Figures 2 and 3). M gene quantification was below the experimental background in both control groups (Figures 2 and 3). In infected NPTr cells, M-vRNA was detected (*P* < 0.001) at 1 h post-infection (hpi) and reached its highest levels 8 and 24 hpi (*P* < 0.001) (Figure 2). Similar results were obtained in PAMs, with detection of M-vRNA as early as 1 hpi (Figure 3); however its level remained slightly lower than that observed in NPTr cells (*Cq* 28 at 1 h for both cell types, and 24 and 18 at 8 h for PAMs and NPTr, respectively). The highest levels of M-vRNA were also observed 8-24 hpi in PAMs (Figure 3).

### Innate immune response evaluation in epithelial cells and alveolar macrophages

Respiratory epithelial cells and PAMs are the first cells encountered by the influenza A virus during an infection in the pig. To explore the innate cellular response, the expression of various transcripts was assessed (Table 1 and Figures 2 and 3). The virus induced the expression of RIG-I mRNA as early as 3 hpi reaching the highest levels of expression at 24 h in NPTr cells (Figure 2). In PAMs, a significant increase (*P* < 0.001) of RIG-I mRNA was observed only after 24 h of infection (Figure 3). Regarding TLR3, TLR7, and TLR8 no statistically significant differences were observed between control and infected NPTr cells (data not shown). However in PAMs, TLR3, TLR7, and TLR8 mRNA expressions were significantly up-regulated by the viral infection (Figure 3). Expression of IFNβ mRNA was clearly increased from 3 hpi in NPTr cells (*P <* 0.05), and from 8 hpi in PAMs (*P <* 0.001) (Figures 2 and 3), and reached its highest level at 24 hpi in both cell types (*P <* 0.001). No significant differences in IFNα mRNA were observed between controls and infected cells, whatever the cell type (Additional file 1). For IFN type III such as IFNλ1, statistically significant increases were observed after 8 and 24 h in NPTr cells but not in PAMs (Figures 2 and 3) while no significant differences were observed for IFNλ3 (data not shown). Regarding the interferon-stimulated genes (ISGs) (Figures 2 and 3), virus-induced mRNA over-expressions (*P* < 0.05 and 0.001) were observed for myxovirus resistance 1 (Mx1), and Mx2 (data not shown), 2′-5′ oligoadenylate synthetase 1 (OAS1), and protein kinase R (PKR) from 3 hpi in NPTr cells and from 8 (Mx1) or 24 hpi (Mx2 – data not shown) in PAMs (*P <* 0.001). The pattern of expression for these genes was similar to that observed for IFNβ transcripts. Among all analyzed SOCS transcripts, only SOCS1 mRNA showed a statistically increased expression after 24 h in both NPTr cells and PAMs (*P <* 0.001). IL-6 (Figure 2) and IL-8 (data not shown) mRNAs were also up-regulated in response to the infection in NPTr cells at 8 and 24 hpi but not in PAMs. Regarding IL-1β and TNFα transcripts, we did not detect any significant differences between conditions in NPTr cells even though some trends of induction by the virus were observed (data not shown), while TNFα mRNA expression was significantly increased (*P* < 0.001) in response to the virus in PAMs after only 3 h of infection (Figure 3).

### Viral infection of precision-cut lung slices

**Figure 4:**
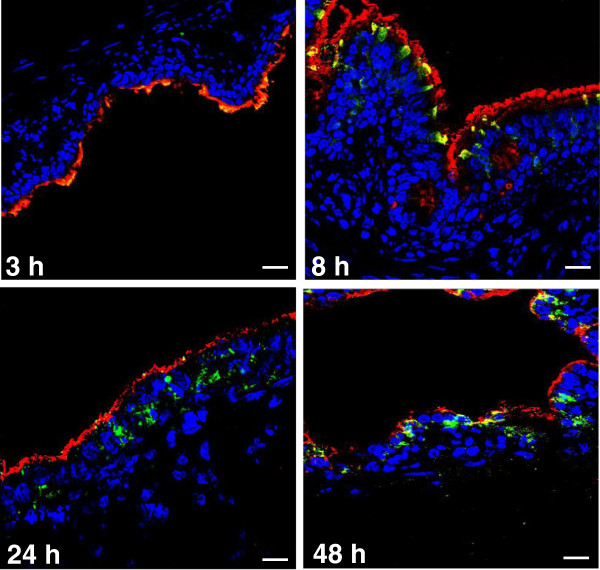
**Immunostaining of infected Precision-cut lung slices (PCLS).** The slices were infected by the H3N2 SIV strain. Cryosections were prepared after 3, 8, 24, and 48 h of infection and image data were collected using a laser-scanning confocal microscope. Infected cells were stained with an anti-nucleoprotein polyclonal antibody (green) for the detection of SIV. Ciliated cells were stained using an anti-beta-tubulin monoclonal antibody (red). Cell nuclei of prepared slides were stained by incubation with 4′,6′-diamidino-2-phenylindole (DAPI, blue). Scale bar = 20 μm.

**Figure 5:**
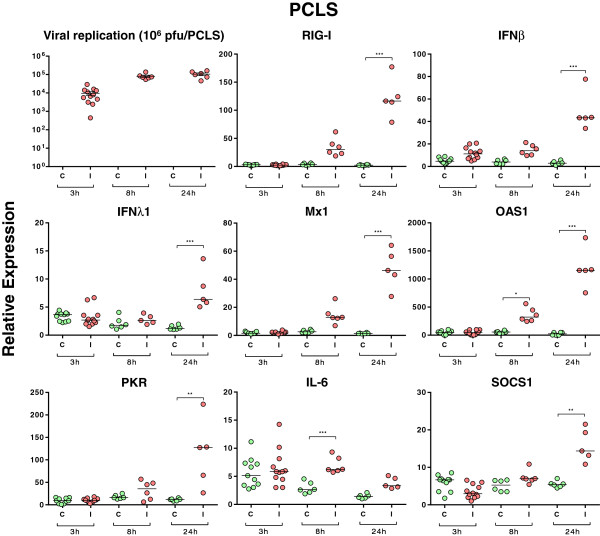
**Relative expression of M-vRNA and of host transcripts in infected precision-cut lung slices (PCLS).** The slices were infected with the H3N2 SIV strain at different time points (3 h, 8 h, and 24 h). Green dots stand for non-infected PCLS and red dots for infected PCLS (minimum one slice/pig for each time point, total 5 pigs, *n* = 5-12 and median value). Comparisons were made using one way ANOVA test and Tukey’s post-test. Differences were considered significant when *P* < 0.05 (*), *P* < 0.01 (**) or *P* < 0.001 (***).

The innate response of PCLS infected with SIV was also analyzed. A minimum of one slice/pig (*n* = 5) was generated at each time point. The viability of the PCLS was verified prior to infection: the ciliary activity of the bronchial epithelium was maintained when analyzed at 24, 48, and 96 h after their preparation (data not shown). Additionally, bronchoconstriction could be triggered by the use of 10^-4^ M methacholine in the four days following slice preparation (data not shown) and subsequently reversed by removal of the drug. These observations provided evidence that porcine PCLS remained viable for up to 96 h under the incubation conditions described in the material and methods section. To monitor viral infection of the slices, cryosections of PCLS were stained with an antibody-targeting SIV nucleoprotein. Only a few infected cells were observed after 3 h of culture with the virus (Figure 4), while the number was considerably increased at 8 hpi. The general architecture of the tissue was progressively altered by the viral infectious process, and after 24 and 48 h a significant lysis of the epithelium was observed (Figure 4). In parallel, viral replication was assessed in the PCLS by qPCR assays targeting the viral M gene segment (Figure 5). M-vRNA was detected only in infected groups, reaching its maximum at 24 hpi (average *Cq*: 22).

### Host transcript expression in precision-cut lung slices

Lung slices offer a more realistic system than cell lines to study the interaction between host and pathogen. To better characterize the innate immune response toward the SIV strain, PCLS were infected and the expression of several host genes involved in the antiviral response was assessed at different time points using qPCR assays (Table 1 and Figure 5). An increase in RIG-I transcripts was observed at 8 h, but it did not become significant until 24 hpi (*P* < 0.001). We did not detect any statistically significant increase in expression of TLR3, TLR7, and TLR8 transcripts post-infection, however some trends potentially indicating a viral induction were observed (data not shown). In response to the virus, IFNβ and IFNλ1 mRNA expression were significantly increased (*P* < 0.001) at 24 hpi (Figure 5) while no significant increase was observed for IFNα and IFNλ3 (Additional file 1 and data not shown). Along with the increased expression of IFN types I and III transcripts, mRNA expression of ISGs Mx1, Mx2, and PKR was also significantly elevated relative to controls after 24 h of infection (*P* < 0.001) (Figure 5 and data not shown). The mRNA expression of OAS1 was significantly increased (*P* < 0.05) after only 8 h of infection (Figure 5). Unlike IL-1β, IL-8, and TNFα, IL-6 transcription was significantly up-regulated 8 h after infection (Figure 5). Overall, except for IFNλ1 and IL-6, similar patterns of transcript expression were observed in vitro in the epithelial cells and alveolar macrophages and ex vivo in the PCLS. Regarding the mRNA expression of SOCS (CISH, SOCS1, and SOCS3), only SOCS1 transcripts were significantly increased at 24 hpi in PCLS (*P* < 0.01) (Figure 5). These findings are similar to those observed with NPTr cells and PAMs and suggest an involvement of SOCS1 in the immune response against SIV.

### Activation of MAP kinases and JAK/STAT pathways in virus-infected epithelial cells

**Figure 6:**
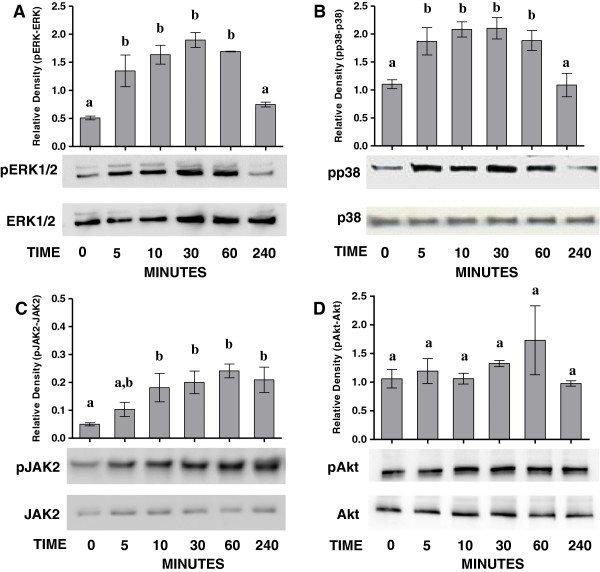
**Western blots in infected NPTr cells.** Western blots for phospho-ERK1/2 (pERK1/2) **(A)**, phospho-p38 (pp38) **(B)**, phospho-JAK2 (pJAK2) **(C)**, and phospho-Akt (pAkt) **(D)** were performed in newborn porcine trachea (NPTr) cells infected with H3N2 SIV at different time points (0, 5, 10, 30, 60, and 240 min). Total ERK1/2, p38 and JAK2 are shown as loading controls and did not change with each condition over time. Data are presented as means ± SEM, (*n* = 3). Results are representative of three independent experiments. Different letters indicate significant differences (one way ANOVA, *P* < 0.05) as compared to control (time 0).

During influenza A infection, various signaling cascades are triggered to counteract pathogen replication and spreading. Many of these signaling pathways are controlled to a certain extent by SOCS proteins [43]. We examined the activation of several signaling pathways involved in pro-inflammatory and antiviral gene expression in response to SIV infection in NPTr cells. MAPK pathways, both ERK1/2 and p38, showed phosphorylations after 5 min of contact with the virus while phosphorylation-mediated activation of JAK2 appeared between 30 and 60 min post-infection (Figures 6A, B, and C). In contrast, activation of the PI3K/Akt signaling pathway was not observed at any time points (Figure 6D); in general, a peak of phosphorylation was observed after 30 to 60 min of infection, followed by a decrease 4 hpi (Figures 6A, B, and C). These results showed that our SIV strain efficiently and rapidly activated MAPK (ERK1/2, p38) and JAK/STAT pathways in NPTr cells.

### Specific inhibition of pathways activated in epithelial cells

**Figure 7:**
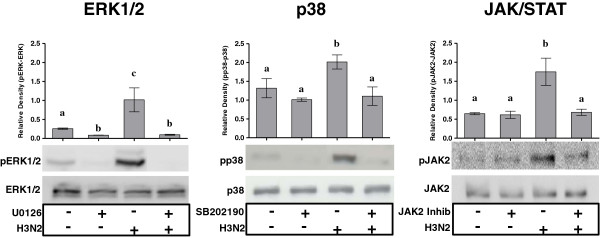
**Western blots in infected NPTr cells in presence or absence of chemical inhibitor.** Western blots for phospho-ERK1/2 (pERK1/2), phospho-p38 (pp38) and phospho-JAK2 (pJAK2) in newborn porcine trachea (NPTr) cells infected with H3N2 SIV at 30 min in presence or in absence of chemical inhibitors UO126 (ERK1/2), SB202190 (p38), and JAK inhibitor (JAK Inhibitor I). The control group was cultured in presence of DMSO, data are represented as means ± SEM, (*n* = 3). Results are representative of three independent experiments. Different letters indicate significant differences (one way ANOVA, *P* < 0.05) as compared to control (without inhibitor and virus).

**Figure 8:**
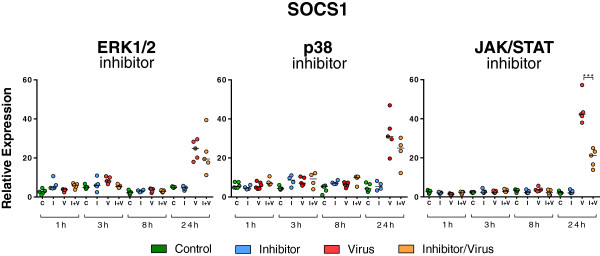
**Relative expression of SOCS1 transcripts in infected NPTr cells in presence of chemical inhibitors.** The relative expression of SOCS1 transcripts was measured in newborn porcine trachea (NPTr) cells infected with H3N2 SIV in presence of chemical inhibitors UO126 (ERK1/2), SB202190 (p38), and JAK Inhibitor I (JAK/STAT) at different time points (1 h, 3 h, 8 h, and 24 h). The control groups were cultured either in the presence of 0.1% DMSO (C, green) or in the presence of inhibitor (I, blue). Infected cells were either untreated (V, red) or inhibitor-treated (I + V, orange). Comparisons were made using one way ANOVA test and Tukey’s post-test (*n* = 5, mean ± SEM). Differences were considered significant when *P* < 0.05 (*), *P* < 0.01 (**) or *P* < 0.001 (***).

**Figure 9:**
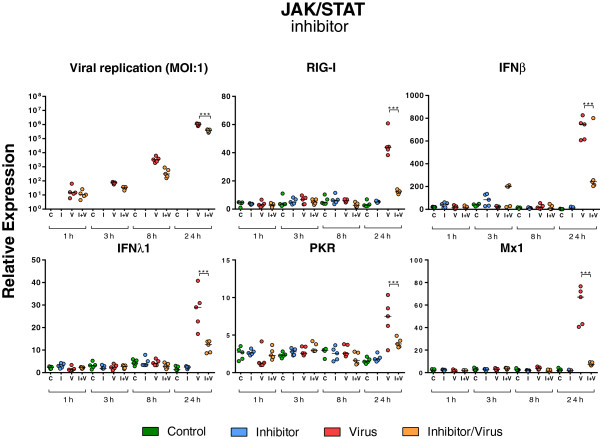
**Relative expression of various viral and host transcripts in infected NPTr cells in presence of JAK Inhibitor I.** The relative expression of various viral and host transcripts was measured in newborn porcine trachea (NPTr) cells infected with H3N2 SIV in presence of JAK Inhibitor I at different time points (1 h, 3 h, 8 h, and 24 h). The control groups were cultured either in the presence of 0.1% DMSO (C, green) or in the presence of inhibitor (I, blue). Infected cells were either untreated (V, red) or inhibitor-treated (I + V, orange). Comparisons were made using one way ANOVA test and Tukey’s post-test (*n* = 5, mean ± SEM). Differences were considered significant when *P* < 0.05 (*), *P* < 0.01 (**) or *P* < 0.001 (***).

To investigate whether the MAPK and JAK/STAT pathways are involved in the regulation of SOCS1 mRNA expression in porcine epithelial cells, NPTr cells were pre-treated with chemical inhibitors targeting these pathways, and the expression of SOCS1 transcripts was assessed at 24 hpi (Figures 7, 8 and 9). One h after the pre-treatment, NPTr cells were infected with the H3N2 strain and phosphorylation was evaluated at 30 min post-infection, corresponding to the peak of activation (Figure 7). NPTr cells pre-treated with inhibitors of ERK1/2 (U0126), p38 (SB 202190) and JAK/STAT (JAK Inhibitor I) in the absence of a virus infection showed basal levels of phosphorylation similar to those observed in control groups. By contrast, pre-treated infected cells displayed a complete abolition of phosphorylation in ERK1/2, p38 and JAK/STAT pathways (Figure 7) lasting more than 24 h (data not shown). In order to determine whether these signaling pathways are involved in the regulation of SOCS1 mRNA expression, the assessment of SOCS1 mRNA expression after inhibitor treatments was performed following the same timing (Figure 8) as in the previous experiment (Figure 2). It was observed that the blockade of ERK1/2 and p38 did not significantly affect SOCS1 mRNA expression after 24 h of infection (Figure 8). However, a statistically significant decrease of SOCS1 mRNA expression was observed in NPTr cells treated with JAK Inhibitor I demonstrating an association between activation of JAK/STAT pathway and SOCS1 mRNA expression in porcine respiratory epithelial cells. To further assess the impact of JAK/STAT pathway-inhibition on virus replication and innate response, the relative expression of M-vRNA and mRNAs for several host proteins was assessed (Figure 9). Viral replication was not significantly modified by JAK Inhibitor I except after 24 h of infection (*P* < 0.001) (Figure 9). Similarly the transcript expression of RIG-I, IFNβ, IFNλ1, PKR, and Mx1 was significantly decreased (*P* < 0.001) under JAK Inhibitor I treatment after 24 h of infection (Figure 9). The inhibition of ERK1/2 and p38 had less impact on viral replication and interferon response (Additional file 2). Viral replication was significantly reduced (*P* < 0.001) at 8 (p38) and 24 hpi (ERK1/2) and OAS1 transcript expression was significantly reduced (*P* < 0.001) with both inhibitors 24 hpi. All together these results demonstrated a link between the JAK/STAT pathway and SOCS1 in pigs.

## Discussion

The innate immune response to swine influenza viruses has been assessed in many previous studies [6,16]. However, to our knowledge most of these studies were carried out in single-cell populations or directly in an animal host. In this study we compared an epithelial cell line (NPTr), alveolar macrophages (PAMs), and precision cut lung slices (PCLS) for their innate immune response to strain A/Swine/Bissendorf/IDT1864/2003 (H3N2). Then, we assessed several signaling pathways and their relation to the innate immune response observed in the NPTr cells.

Viral replication was higher in NPTr cells than in PCLS and PAMs. This is in agreement with the current knowledge of influenza pathogenesis. Indeed, it is well known that the main target of the virus is epithelial cells [12,15] and NPTr is a cell line consisting of tracheal epithelial cells [18]. This was also confirmed by the immune-staining of the PCLS, where many ciliated epithelial cells were observed in the associated bronchi. In the three systems used in this study, a strong and progressive innate immune response was triggered by the virus. The response in NPTr cells and PCLS was very similar except for a trend toward TLR induction in the latter. This could be explained by the presence of various cell types such as macrophages and/or dendritic cells in this tissue [6,14]. Moreover, observed discrepancies between NPTr cells and PCLS may also be related to the less controlled percentage of cells infected in the PCLS (MOI cannot be determined) and to the potential resistance of a significant proportion of the various cell types to SIV infection. Our study further demonstrated the value of using PCLS in the study of pig/SIV interactions. RIG-I transcription was induced by the virus in NPTr cells, PAMs, and PCLS while TLR3, TLR7 as well as TLR8 transcription were significantly induced only in PAMs. This result was a little bit surprising since it has been observed recently that airway epithelial cells mostly use surface and endosomal TLR3 to detect influenza virus and to initiate IFN production [44]. Following recognition of the virus, interferon responses were triggered. In NPTr cells and PCLS we observed mostly IFNβ and IFNλ1 transcript expression in response to the virus while in PAMs, IFN type III was not significantly induced. Regarding the expression of IFNα transcripts, no significant differences between infected and non-infected cells/tissues were observed. This result is surprising as IFNα transcripts were previously detected in PAMs infected by another strain of SIV [45]. In that study IFNα mRNA was detected earlier than IFNβ mRNA. The use of qPCR primers targeting IFNα 1-17 may have masked differences in the expression of transcripts associated to a specific IFNα gene. It is known that significant variations in the expression of the various IFNα transcripts upon infection can be observed [46]. Recently, it has been demonstrated that type III IFN is the predominant IFN produced by the airway epithelium [44]. Our data showed the increased production of mRNAs encoding both type I and type III IFNs in epithelial cells. The absence of type III IFN induction in PAMs supports the idea of a predominant role of type III IFN in a specific cell type such as epithelial cells as suggested by Ioannidis et al. [44]. In response to IFN, ISG (Mx1, Mx2, OAS1, PKR) transcripts were identified in the three systems. This observation was particularly clear with the NPTr cells probably because of their nature (epithelial *versus* alveolar macrophages) and their high purity in comparison to primary epithelial cells located in the slices. These two features also probably account for the quicker and higher response observed in NPTr cells when compared to PAMs and PCLs. Because of their role in the regulation of immune response [47,48] and in influenza pathogenesis [49], we looked at the expression of SOCS transcripts in response to SIV in the three systems. Twenty-four hpi SOCS1 transcripts were up-regulated in the three systems. This observation confirmed our previous study postulating the inducible nature of SOCS1 in porcine lungs [33]. Instead of SOCS1, a role for SOCS3 has been recently identified in pigs [50]. The authors showed that the lower susceptibility of pigs to contemporary Eurasian highly pathogenic avian influenza (HPAI) H5N1 virus infection is linked to SOCS3 induction. Under their conditions, swine epithelial cells were expressing SOCS3 proteins in response to both human H1N1 virus and HPAI H5N1 virus [50]. In another study, it was demonstrated that influenza A virus induced NF-κB dependent SOCS3 gene expression in human epithelial cells, suppressing type I IFN response through the JAK/STAT pathway [51]. In our conditions, SOCS3 was never significantly induced in response to H3N2 SIV. It remains to be shown whether these differences are due to different experimental parameters (e.g. virus strain and timing of measurements) or potential discrepancies between RNA levels and protein expression.

In NPTr cells, the Akt signaling pathway was not significantly induced, while we did observe the triggering of MAPK (ERK1/2 and p38) and JAK/STAT pathways. The absence of Akt activation was unexpected, since this signaling pathway was shown to be activated by influenza A virus, probably favoring viral replication [32,52]. However, we assessed different pathway activations early in the infection process, and the protocol used in our study did not allow for the observation of Akt activation. The activation of JAK/STAT pathway has been reported in other species [13]. This pathway controls type I IFN response. JAK/STAT signaling induced formation of a trimeric transcription factor ISGF3, consisting of STAT1, STAT2, and interferon regulatory factor 9 (IRF-9), which regulates the expression of several ISGs [13]. Four MAPK cascades have been described including two isoforms of the ERK1/2, the Jun-terminal kinase (JNK), p38, and ERK5 [52,53]. The signaling pathways convert extra- and intracellular signals into cellular responses, regulating cell activation, differentiation, immune responses, and proliferation [52,53]. In our study we assessed the activation of ERK1/2 and p38, and activation of both cascades was observed. p38 predominantly responds to stress conditions and pro-inflammatory cytokines, whereas ERK1/2 senses mitogenic stimuli [52,53]. MAPK signaling pathways and their role in the regulation of innate response and viral replication have not been well studied in porcine cells infected by influenza virus. However, a recent study showed a robust activation of ERK1/2 in influenza virus-infected swine macrophages [54] and demonstrated that the induction of RIG-I and MDA-5 depends on the activation of ERK1/2 and JNK in these cells. Interestingly and similar to our findings, these authors did not observe any IL-1β induction in response to the tested influenza virus [54]. Unlike IL-1β expression, TNFα was clearly induced early at 3 hpi.

We also investigated to what extent inhibitors of the different pathways could impact SOCS1 transcription. Only JAK/STAT pathway inhibition was accompanied by a significant decrease in the expression of SOCS1 transcripts. This observation confirms the strong association of SOCS (particularly CISH, SOCS1, and SOCS3) with the JAK/STAT pathway. Nevertheless, in our conditions the association was only clear for SOCS1 and not for the two other SOCS family members. To further assess the impact of JAK/STAT pathway inhibition, we then looked at the expression of transcripts accounting for viral replication, viral recognition and interferon response. The treatment with JAK Inhibitor I was accompanied by a significant decrease in viral replication (as assessed by quantification of M-vRNA) and by a diminished expression of RIG-I, IFNβ, IFNλ1, PKR, and Mx1 transcripts 24 h post-infection. The decrease in viral replication remains unexplained, although it could be linked to the presence of more IFN in the culture supernatant consecutive to an inhibition of SOCS1. However, the lower level of expression of IFN transcripts argues against this hypothesis. In another set of experiments using another SIV strain (unpublished results) we used pJAK2(1001-1013) inhibitor [55,56] and obtained similar results in both epithelial cells and macrophages. The viral replication was significantly reduced, as were the transcriptions of IFN and ISGs. Regarding the inhibition of MAPK pathways, we observed a decrease in viral replication as well as a decrease in the interferon response. Similarly, a study by Pinto et al. [57] demonstrated in human epithelial cells that the inhibition of MEK in the Raf/MEK/ERK pathway resulted in reduced virus titers and lower expression of pro-inflammatory cytokines [57,58]. In another study on HPAI H5N1 virus in mice [59] it was also shown that the inhibition of p38 greatly reduced virus-induced cytokine expression concomitant with reduced viral titers.

In conclusion, our study showed the advantage of using various cellular and tissue systems in the study of the innate immune response to influenza virus. The kinetic approach enabled a comparative assessment of the innate immune response in the main targets of the virus and brought insights to viral triggering of major signaling pathways in NPTR cells. Our data suggests a link between SOCS1 and the JAK/STAT pathway in porcine respiratory epithelial cells. The inhibition of the JAK/STAT pathway using JAK Inhibitor I clearly reduced the response of IFN types I and III and the induction of SOCS1 at the transcript level in NPTr cells. Similarly the inhibition of MAPK pathway reduced viral replication and interferon response. Further investigations are required to understand precisely the role of SOCS in pigs. A better understanding of the host innate immune response and its underlying regulation mechanisms will help identify strategies for effective control of SIV.

## Competing interests

The authors declare that they have no competing interests.

## Authors’ contributions

MDO carried out most of the experiments, participated in the analysis of the data and drafted the manuscript. SM helped MDO to carry out the experiments. DP participated actively to PCLS experiment. CR was involved in the Western blot preparation and analysis. MO and DS helped for the preparation of the virus, titration and cell culture. DM, GS, GH, MB, and JD participated in the design of some experiments and helped to draft the manuscript. FM conceived the study, actively participated in its design and coordination, analyzed the data, drafted and revised the manuscript. All authors read and approved the final manuscript.

## Supplementary Material

Additional file 1 **Relative expression of IFNα (1-17) transcripts in infected NPTr cells, PAMs and PCLS.** The cells and tissue were infected with the H3N2 SIV strain at different time points (1 h, 3 h, 8 h, and 24 h). Green dots stand for non-infected cells and tissue. Red dots stand for infected cells and tissue (NPTR cells: individual values (dots) and median value (bar), *n* = 6 wells per condition; PAMs: minimum one slice/pig for each time point, total 5 pigs, *n* = 5-12 and median value; minimum one slice/pig for each time point, total 5 pigs, *n* = 5-12 and median value). Comparisons were made using one way ANOVA test and Tukey’s post-test. Differences were considered significant when *P* < 0.05 (*), *P* < 0.01 (**) or *P* < 0.001 (***). Click here for file

Additional file 2 **Relative expression of various transcripts in infected NPTr cells in presence of MAPK inhibitors.** The relative expression of various viral and host transcripts was measured in newborn porcine trachea (NPTr) cells infected with H3N2 SIV in presence of ERK1/2 and p38 inhibitors at different time points (1 h, 3 h, 8 h, and 24 h). The control groups were cultured either in the presence of 0.1% DMSO (C, green) or in the presence of inhibitor (I, blue). Infected cells were either untreated (V, red) or inhibitor-treated (I + V, orange). Comparisons were made using one way ANOVA test and Tukey’s post-test (*n* = 5, mean ± SEM). Differences were considered significant when *P* < 0.05 (*), *P* < 0.01 (**) or *P* < 0.001 (***). Click here for file
