# Supplementary figures and images for: Innate immune response to a H3N2 subtype swine influenza virus in newborn porcine trachea cells, alveolar macrophages, and precision-cut lung slices
Source: Vet Res. 2014 Apr 9;45(1):42. doi: 10.1186/1297-9716-45-42 (PMC4021251; doi:10.1186/1297-9716-45-42)

## Slide 1
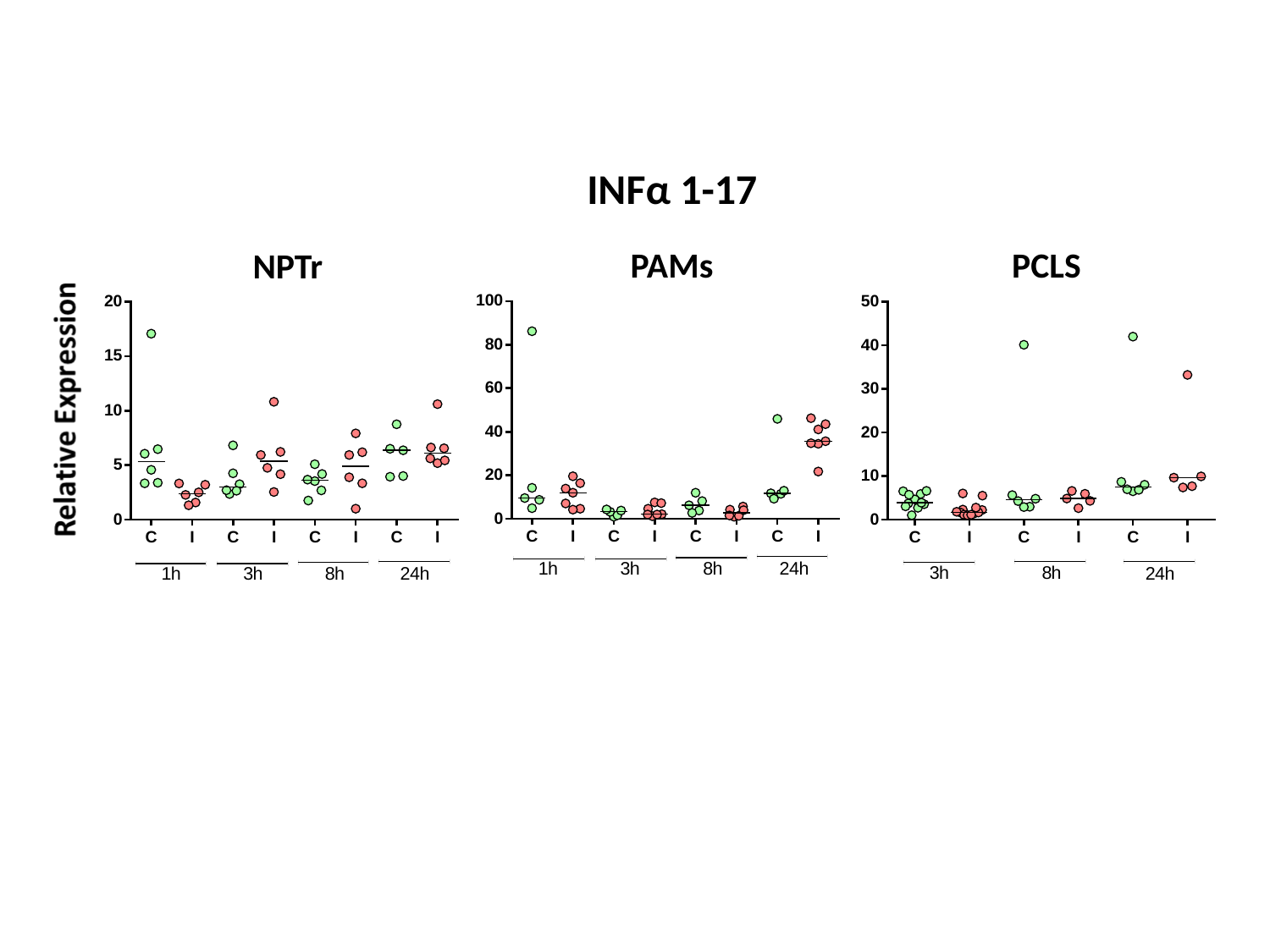

INFα 1-17
PCLS
PAMs
NPTr

Supplement: Additional file 1 — Relative expression of IFNα (1-17) transcripts in infected NPTr cells, PAMs and PCLS. The cells and tissue were infected with the H3N2 SIV strain at different time points (1 h, 3 h, 8 h, and 24 h). Green dots stand for non-infected cells and tissue. Red dots stand for infected cells and tissue (NPTR cells: individual values (dots) and median value (bar), n = 6 wells per condition; PAMs: minimum one slice/pig for each time point, total 5 pigs, n = 5-12 and median value; minimum one slice/pig for each time point, total 5 pigs, n = 5-12 and median value). Comparisons were made using one way ANOVA test and Tukey’s post-test. Differences were considered significant when P < 0.05 (*), P < 0.01 (**) or P < 0.001 (***). [file 1297-9716-45-42-S1.ppt]

## Slide 1
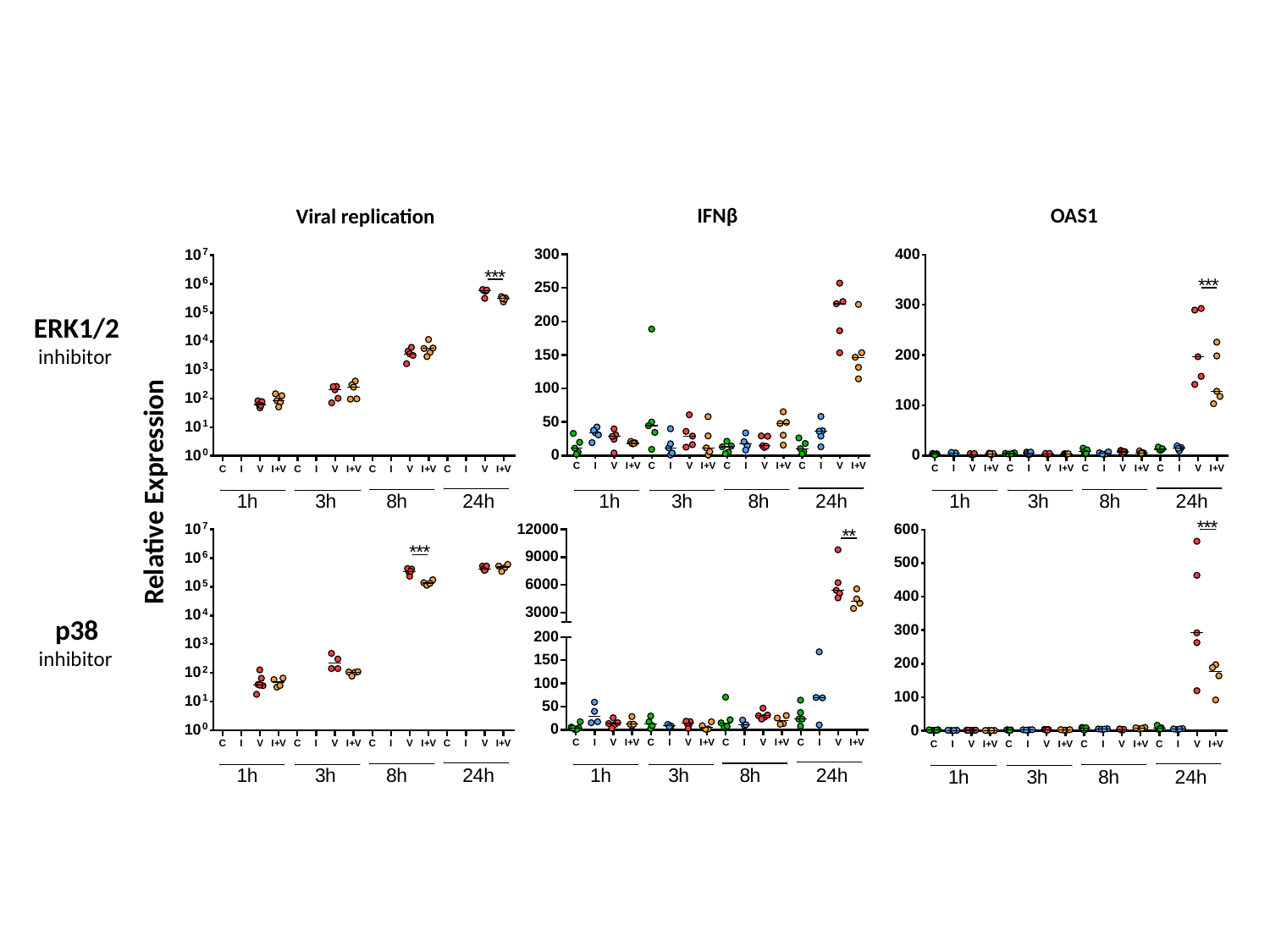

IFNβ
OAS1
Viral replication
ERK1/2
inhibitor
Relative Expression
p38
inhibitor

Supplement: Additional file 2 — Relative expression of various transcripts in infected NPTr cells in presence of MAPK inhibitors. The relative expression of various viral and host transcripts was measured in newborn porcine trachea (NPTr) cells infected with H3N2 SIV in presence of ERK1/2 and p38 inhibitors at different time points (1 h, 3 h, 8 h, and 24 h). The control groups were cultured either in the presence of 0.1% DMSO (C, green) or in the presence of inhibitor (I, blue). Infected cells were either untreated (V, red) or inhibitor-treated (I + V, orange). Comparisons were made using one way ANOVA test and Tukey’s post-test (n = 5, mean ± SEM). Differences were considered significant when P < 0.05 (*), P < 0.01 (**) or P < 0.001 (***). [file 1297-9716-45-42-S2.ppt]
